# Melting Point and Crystal Growth Kinetics of Metals and Metal Oxides Using Reactive Force Fields: The Case of Aluminum and Alumina

**DOI:** 10.1021/acs.jctc.4c00628

**Published:** 2024-09-05

**Authors:** Hao Zhao, Fernando Bresme

**Affiliations:** † Department of Chemistry, Molecular Sciences Research Hub, Imperial College, London W12 0BZ, U.K.; ‡ State Key Laboratory of Multiphase Flow in Power Engineering, Xi’an Jiaotong University, Xi’an, Shaanxi 710049, China

## Abstract

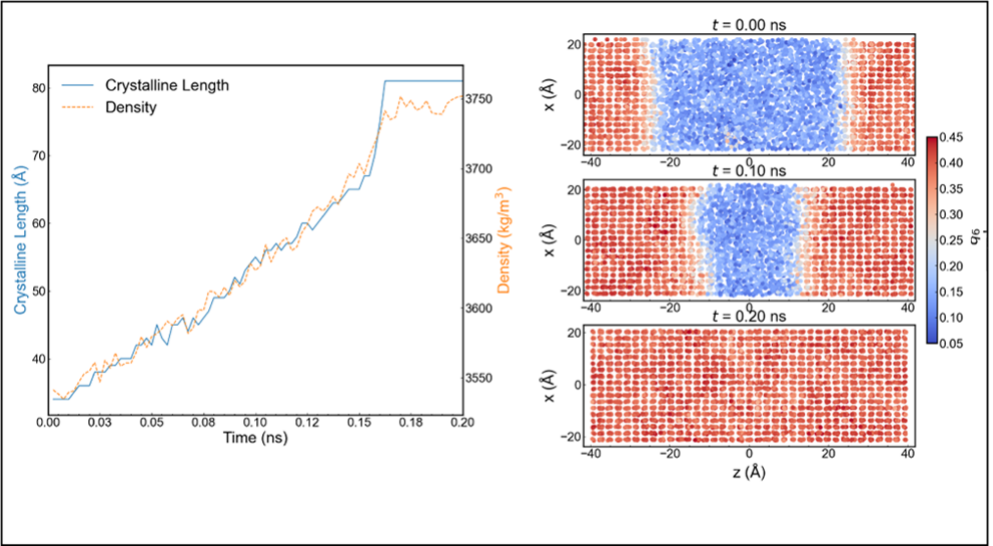

Alumina and aluminum are strategic materials employed in energy applications, with metal aluminum being interesting in phase change material applications. Therefore, the theoretical description of the thermophysical properties of these materials represents an important objective. Here, we investigate the liquid–solid coexistence properties of aluminum and alumina using a state-of-the-art reactive force field (ReaxFF) and molecular dynamics simulations. Aluminum features ultrafast crystal growth, which enables the direct determination of its melting temperature via direct coexistence simulations (858 ± 2 K). However, at standard pressure, alumina is easily trapped in a glass state, preventing the application of the direct coexistence method. We demonstrate that direct coexistence can be used at high pressures above 2 GPa, where alumina features a higher melting temperature, and the liquid–solid interface exhibits enhanced dynamics. Our approach opens a route to obtain the melting temperature of ReaxFF alumina at standard pressure (1670 ± 10 K) and, more generally, a viable method for calculating the melting point of metal oxides via direct coexistence simulations. We further investigated the dynamics of crystal growth of the solid–liquid aluminum and alumina interfaces.

## Introduction

Alumina (Al_2_O_3_) is an important material that can form naturally on the surface of aluminum (Al) when the latter comes into contact with air. The thin alumina film (2–4 nm)^1^ on the surface of aluminum provides effective protection for aluminum from corrosion. This protective and self-healing layer^2^ ensures the longevity and durability of aluminum and allows it to be widely used across various industries,^3^ including aerospace^4^ and electronics.^5^ Given the high reactivity of Al and the inert nature of Al_2_O_3_, the Al/Al_2_O_3_ metal–ceramic interface has attracted significant attention in various studies, including wetting,^6−8^ sintering,^9^ and crystal growth.^10^ Among the theoretical studies employing molecular dynamics (MD) simulations, such as Al/α–Al_2_O_3_ interface structure investigations,^11,12^ alumina is commonly treated as a solid phase due to its high melting temperature (*T*_m_ = 2314 ± 7 K).^13^ Simulations focusing on aluminum oxidation reported that freshly formed alumina can melt during the oxidation of aluminum nanoparticles.^14^

The melting point of alumina varies with the system size, becoming relevant at nanometer length scales. Dreizin et al.^15^ reported depression of the melting point by 200–400 K for a 3–5 nm natural alumina film^1^ using theoretical analyses. The melting point of bulk alumina provides a fundamental thermodynamic property to investigate multiphase processes, such as aluminum oxidation, which has been investigated recently using computer simulations.^16,17^ However, previous studies have not focused sufficiently on the alumina melting properties. Indeed, there are currently no accurate estimates of alumina using ReaxFF simulations, despite the relevance of this force field to describe reactivity in metal oxides.

The dynamics of crystal growth of metals is important in phase change material applications. Previous studies on crystal growth provided important insights into the microscopic mechanisms determining the ultrafast crystal growth of metals,^18^ with crystal growth rates exceeding 100 m/s. Fast crystal growth rates for atomistic solids were also reported in a recent nonequilibrium molecular dynamics/continuum modeling investigation.^19^ The latter study provided evidence for a slow crystal growth rate under thermal gradient. The dynamics of crystal growth of alumina is also important but very challenging to investigate at a standard pressure. Experimental metal oxide growth rates typically range from 10^–5^ to 10^–9^ m/s.^20^

The reactive force field (ReaxFF)^21,22^ interatomic potential, with its capability of describing the changes of chemical bonds through the distance-based and element-based bond-order functions, has emerged as a preferred method to account for the reactivity of aluminum and alumina surfaces. This force field has been parameterized to investigate a wide variety of problems connected to aluminum, such as the oxidation of aluminum nanoparticles,^21,23^ the wetting transition in the Al/Al_2_O_3_ interface,^7^ carbon coating on aluminum nanoparticles,^24^ or aluminum water reactions.^25^ Although ReaxFF has been used in molecular dynamics simulations to investigate the kinetics of aluminum chemical reactions at the nanosecond level, the study of the thermophysical properties of aluminum and aluminum compounds using ReaxFF has received little attention to date. Specifically, only the melting point of aluminum has been reported using ReaxFF. These investigations include aluminum nanoclusters (800 K^26^), aluminum nanoparticles (440–600 K for diameters ranging from 2.0 to 4.0 nm^27^), and bulk aluminum (860 K^11^). The estimated melting temperature of bulk ReaxFF is close but lower than the experimental value of 933 K.^28^

The lack of simulations on the melting point of alumina is primarily attributed to its slow crystal growth rate.^20^ As noted above, metal growth rates can reach up to 10^2^ m/s and are therefore amenable for investigation using nanosecond molecular simulations. However, the slow growth rate of metal oxides (see above) greatly exceeds the current capabilities of atomistic molecular dynamics methods. Furthermore, the dependence of glass-transition temperature (*T*_g_) on the cooling rate^29^ also restricts the direct observation of crystallization. The limitation of simulation nanosecond time scales requires a high cooling rate, thereby increasing the glass-transition temperature (*T*_g_). Experimental results indicate that cooling rates of ∼10 K/ps are suitable for observing aluminum crystallization,^30,31^ while alumina requires a much lower cooling rate of 10^–10^ K/fs to prevent its vitrification.^32^ While the mechanism for the rapid crystallization of Al is understood as barrierless crystal growth kinetics,^18^ the slow crystal growth kinetics of alumina limits the ability to access atomic-level insights from direct molecular dynamics simulations.

We hypothesize that increasing the pressure can speed up the slow kinetics of crystal growth, as this would lead to a concomitant increase in the melting temperature, as predicted by Clapeyron’s equation. Indeed, recent theoretical/experimental studies support this hypothesis, as those studies demonstrated that high-pressure conditions increase the crystallization speed of supercooled dimethyl phthalate.^33^ We test this hypothesis in this work by analyzing the effect of pressure on the melting temperature of alumina. Advancing the discussion below, we find that pressure increases the crystal growth kinetics, enabling the estimation of the standard melting temperature of alumina by extrapolating high-pressure data to 1 bar pressure.

Here, we report reactive force field MD simulations of the melting point of bulk alumina Al_2_O_3_ and aluminum (Al) using the ReaxFF.^21,22^ We employ the direct solid–liquid coexistence method to obtain the melting temperatures of these compounds. To the best of our knowledge, this study reports the first calculation of the melting point of bulk alumina by utilizing the ReaxFF force field. We further analyze and quantify the crystal growth kinetics of aluminum and alumina. Overall, our simulations provide information about the energy barriers controlling the kinetics of crystal formation at the nanometer scales. This theoretical study demonstrates the capabilities of high-pressure simulations to quantify the melting temperature of complex metal oxides. Furthermore, we assess the accuracy of ReaxFF to describe the melting processes in metals and metal oxides, providing fundamental thermophysical properties for future modeling of the reactivity of the aluminum/alumina interface.

## Methodology

### ReaxFF Force Field

The potential energy function in the ReaxFF can be described as^21,22^
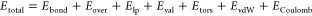
1where *E*_total_ corresponds to the total energy of the system, *E*_bond_ is the energy involved in the formation of bonds between atoms, *E*_over_ is the over/undercoordination correlation energy, *E*_lp_ is the lone pair energy, *E*_val_ is the valence angle energy, *E*_tors_ is the torsion energy, *E*_vdW_ is the van der Waals energy, and *E*_Coulomb_ is to the Coulomb energy. ReaxFF introduces the bond-order (BO) concept to calculate and characterize the chemical bond interactions where the BO is a function of atom distances and atom element types. Further details can be found in refs (21,23). The parameter sets of the ReaxFF employed here were optimized in ref (14) using the one-parameter parabolic extrapolation against density functional theory (DFT) training sets.^34,35^ The training data include equations of state, adsorption and decomposition energies, and bond dissociation/angle distortion energies. The first Al/O parameter set is developed by Zhang et al.^7^ in the study of the Al/α–Al_2_O_3_ interface. Later, Hong and van Duin^14^ updated the Al/O parameter set (Al/O-2015) and validated its accuracy in describing aluminum oxidation. Besides, the Al/O-2015 parameter set remained unchanged and was used by Hong et al. to develop the Al/C/H/O force field set.^24^ In this work, the Al/O-2015 set was employed. Additionally, compared with the embedded-atom model (EAM), which lacks the capability to simulate charge transfer or chemical bond formation and breaking, the ReaxFF force field offers significant advantages. ReaxFF, by including polarization effects, charge transfer, and reactive processes, provides a more comprehensive description of the complex microscopic interactions at play, especially in heterogeneous environments such as the liquid–solid interfaces explored in our work.

### Simulation Setup

**Figure 1:**
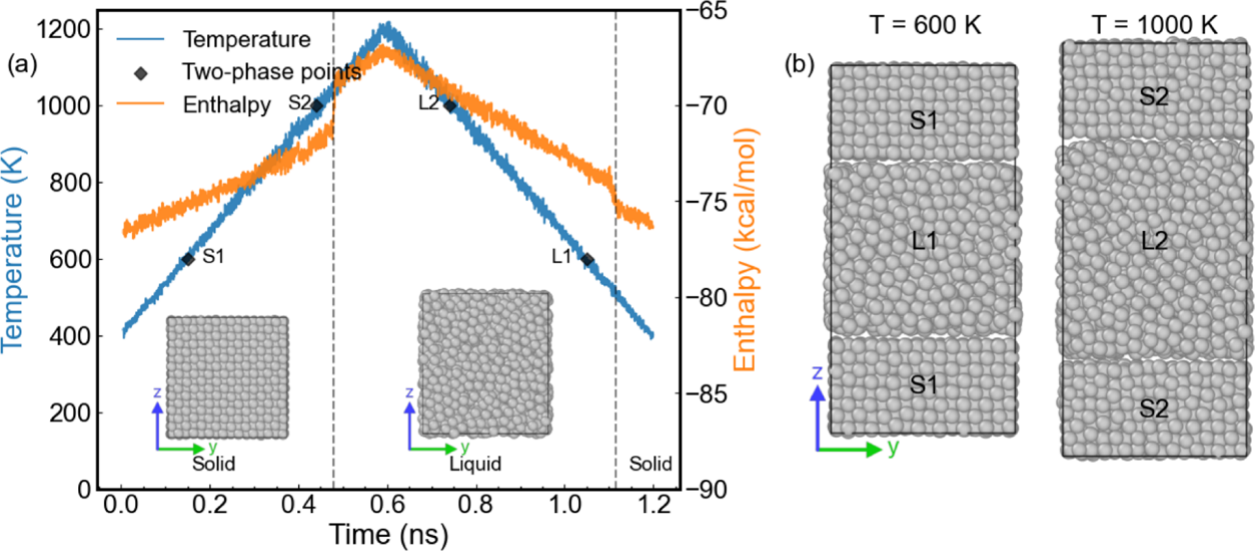
Simulation setup to perform direct coexistence computations of aluminum. (a) Single-phase preparation and (b) initial configurations of coexistence at 600 and 1000 K. The bulk phase was heated from 400 to 1200 K, followed by subsequent cooling to 400 K (represented by the blue line). The scattered data points, denoted as “S1,2” for solid and “L1,2” for liquid, indicate the configurations used to build the solid–liquid coexistence systems at different temperatures. The distinction between solid and liquid phases can be seen in the discontinuous change of the enthalpy (see vertical lines crossing the enthalpy curve (orange)).

The direct coexistence simulations were prepared by combining separately relaxed supercooled liquids and superheated solids, both with the same specified temperature, as shown in Figure 1. The supercooled liquids and superheated solids were obtained from single-phase bulk heating and cooling processes. The initial configurations of bulk aluminum and alumina were constructed from crystallography data.^36,37^ The trigonal alumina structure was converted to a cubic cell using the transformation package of pymatgen.^38^ Subsequently, supercell operations were employed to build a 32 × 32 × 32 Å^3^ simulation box containing 2048 atoms for aluminum and a 43 × 33 × 39 Å^3^ box with 6480 atoms for alumina. Each atom was initially assigned a velocity using a Gaussian velocity distribution, targeting 400 and 1000 K for aluminum and alumina, respectively. These temperatures correspond to approximately half of the experimental melting temperature. Subsequently, the solid was heated to generate the liquid phase, reaching temperatures of 1200 K for aluminum and 3000 K for alumina, followed by a cooling-down process back to temperatures of 400 K for the solid phase of Al and 1000 K for the glass phase of Al_2_O_3_. During the heating/cooling cycle, the configurations of superheated solids and supercooled liquids were stored every 10 K by dumping the atomic coordinates, velocities, and charges. Our single-phase simulation preparation consisted of consecutive procedures (see Figure 1): (1) 10,000-step isothermal–isobaric (NPT) run involving a temperature change of 10 K for heating or cooling, (2) a 10,000-step equilibrium NPT run, and (3) a 10,000-step isothermal–isovolumetric (NVT) for sampling with a dumping frequency of 100 time steps. The thermostat and barostat were implemented using the Nosé–Hoover method^39,40^ with damping constants of 50 fs for temperature and 250 fs for pressure. The average heating/cooling rate was 2 K/ps, a sufficiently slow rate to prevent excessive heating^41^ when simulating the ReaxFF force field. Finally, the liquid–solid interfaces were built by joining liquid and solid slabs, with the solid faces corresponding to the (100) surface for aluminum and the (0001) surface for alumina. We introduced a small 2 Å vacuum spacing between the liquid and solid phases to facilitate the relaxation of the initial configuration. Our strategy to build the liquid–solid configurations involves the relaxation of the liquid and solid phases separately and has two advantages: first, it allows the bulk phase with more relaxation time; second, it enables the execution of parallel coexistence simulations at different temperatures, which is the most time-consuming step. The key aspects of our approach are summarized in Figure 1.

The direct coexistence simulations were conducted for 1 ns under the NPT ensemble, with two independent barostats: one for the *z*-direction (perpendicular to the liquid/solid interface) and one for the *xy*-coupled directions to ensure that the interface was simulated at the target pressure. A time step of 0.25 fs was used in all of the simulations, as required by the dynamic bond-order update algorithm of the ReaxFF. The temperature damping time was 200 time steps (equivalent to 50 fs), and the pressure dumping time was 1000 time steps (250 fs). The simulation results were averaged from 10 replicas with different initial velocity seeds. All MD simulations were conducted using the LAMMPS program^42^ (version 2 Aug 2023) with the ReaxFF package.^43^ Atomic configurations were visualized using the OVITO software.^44^

### Computational Approach to Calculate Melting Points

The Lindemann index^45^ was used to monitor the location of the phase transition as a function of temperature. The internal-energy-based method can be used to identify the location of bulk melting points. In addition to identifying bulk melting points, the Lindemann index offers critical insights into local properties in a material, and it is particularly suitable for analyzing interfacial phenomena and therefore provides a more consistent metric to analyze our simulations, as they involve explicit interfaces. The system-averaged and time-averaged Lindemann indices^45^ are defined as
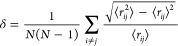
2where *N* is the number of atoms, ⟨ ⟩ denotes the time average, and the term *r*_*ij*_ is the folded distance between atom *i* and atom *j*, adjusted to account for the periodic boundary condition (PBC). In our calculations, using PBCs influences the values ⟨*r*_*ij*_ ⟩ in eq 2 as the largest interparticle distance is half the box length. Consequently, the Lindemann index calculated in this way features an increase in its value as the sampling time increases. The increase is larger in the liquid phase due to the enhanced atom mobility. Therefore, we identify the phase transition by observing the jump in the Lindemann index, rather than depending on a fixed threshold criterion.^46^

The melting points were estimated by investigating the thermal stability of the coexisting systems at different temperatures. The per-atom bond orientational order parameter *q*_6_^′^,^47,48^ a well-defined and robust structure metric, was used to monitor the crystallization or melting state of the system. A small *q*_6_^′^ (typically lower than 0.2) indicates the presence of neighboring atoms in a disordered configuration, typically observed in a liquid state. Conversely, a large *q*_6_^′^ (typically >0.4) means a higher degree of order, which is associated with the solid state. The *q*_6_^′^ is a weighted Steinhardt order parameter, and it is defined as
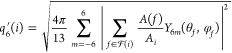
3where *Y*_6*m*_ is the spherical harmonic with the components of degree 6, *A*(*f*) is the surface area of the Voronoi cell facet *f* belonging to the Voronoi cell boundary  from atom *i*, and . The Voronoi tessellation is calculated with the Voro++ library.^49^ In the case of aluminum, we used all of the Al atoms in our analysis. For the Al_2_O_3_ system, the oxygen atoms were initially excluded, and only the aluminum atoms were included in quantifying the local ordering. We used the *q*_6_^′^ parameter to quantify the length of the crystal slab *l* along the direction of the crystallization/melting front (*z*-direction). A per-layer *Q*_6_^′^(*z*) parameter was calculated from the per-atom average of *q*_6_^′^ by projecting the atomic results on planes perpendicular to the direction of motion of the crystallization/melting front
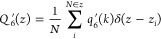
4where *N* is the number of atoms with *z*-coordinates within the range of(*z*–d*z*/2,*z* + d*z*/2), and d*z* was chosen as 0.01 nm. The crystal slab length, *l*, is then given by consecutive layers, whose order parameter exceeding 0.95 of the solid-state value
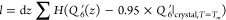
5where the sum runs over the bins located at coordinate *z*, *H*(*x*) is the Heaviside step function, and d*z* represents the length of a single layer. The value of 0.95 was considered following the ref (18) to account for the thermal fluctuation.

For the aluminum system, which features an ultrafast crystal growth rate,^18^ the complete melting and crystallization process in the explicit liquid–solid interface was followed by monitoring the crystal slab length *l*. The melting point was determined as the average of two temperatures corresponding to the slowest rates of increase or decrease in parameter *l*, derived from temperature series simulations conducted with a scan step of 1 K.

The dynamic scan range method was used to improve the simulation efficiency. This method targets the transition temperatures of the superheated solid and supercooled liquid and therefore the hysteresis cycles associated with a heating/cooling process. This method can be readily applied to metals as these feature rapid crystallization from the supercooled liquid state. To implement the dynamic scan range method, we first choose the temperature in the range (*T*_m0_ – 100,*T*_m0_ + 100) with a scan step of 10 K, followed by the temperature range exhibiting the slowest crystallizing and melting rates, with a scan step of 1 K. The initial guess of the melting point (*T*_m0_) was obtained using the empirical formula^50^
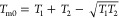
6where *T*_1_ and *T*_2_ are the transition temperatures of the superheated phase and supercooled phase, respectively, which were determined using the Lindemann index as discussed above. For alumina, it is possible to observe the transition of the superheated solid to the melt. However, as we show below, upon cooling and due to the fast cooling rate employed in the simulations, the supercooled liquid state can fall easily into a high-temperature glass state of 1329 K. While we found *a posteriori* that this glass-transition temperature was close to the actual melting temperature of ReaxFF alumina, the direct solidification of the supercooled alumina at standard pressure conditions was not observed in the simulations. However, as discussed in the Results and Discussion section, the crystallization can be readily observed upon cooling at pressures above 2 GPa. We found that due to the higher melting temperature at high pressure, the kinetics of crystal growth increases significantly relative to the standard pressure conditions.

An integral part of our method to obtain the melting point of the oxide compound is the calculation of the crystal growth rate. To model the simulation results of the crystal growth rate, *u*, we used as a starting point the Wilson and Frenkel equation,^51,52^ which has been employed by Broughton et al.^53^ to investigate the solidification of simple atomistic solids
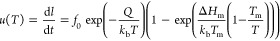
7where *T* is the system temperature, *f*_0_ = 6*aD*_0_/Λ^2^ is a fitting parameter, with *a* being the thickness of the interface, Λ is the mean free path associated with the phase change process and *D*_0_ for the diffusion prefactor, *Q* is the activation energy for diffusion in the liquid, Δ*H*_m_ = *H*_liquid,*T* =*T*_m__ – *H*_solid,*T* =*T*_m__ is the enthalpy of melting, *T*_m_ is the melting point, and *k*_B_ is the Boltzmann constant. In eq 7, we use the approximation, Δμ = μ_l_ – μ_s_ ≈ Δ*H*_m_ (*T*_m_ – *T*)/*T*_m_, which has been shown to reproduce the simulation data of homogeneous crystallization of metals.^54^

In our work, a positive moving rate indicates solidification of the system, and a negative value means melting. For the simulations under standard pressure conditions, we employed the rate of melting only.

## Results and Discussion

### Melting Points of Aluminum

**Figure 2:**
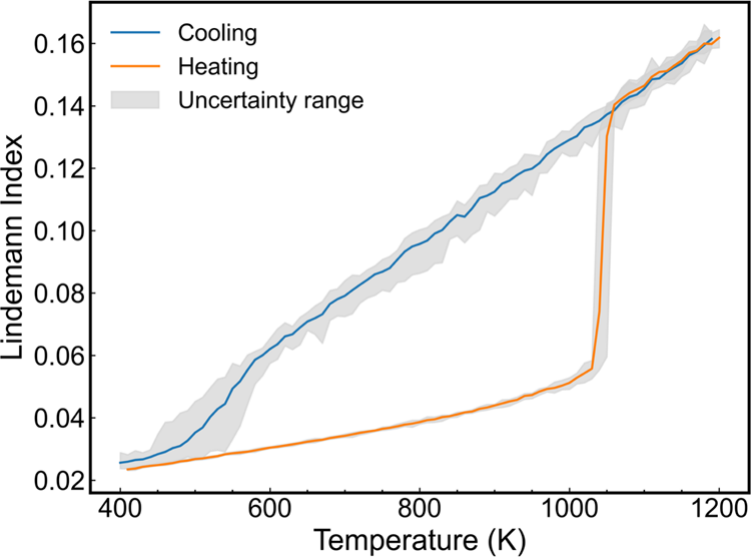
Lindemann index of bulk aluminum. The shaded regions represent the uncertainty associated with the Lindemann index. These uncertainties were calculated using results obtained from 10 independent trajectories, each lasting 1.2 ns. The orange and blue lines represent the heating and cooling cycles, respectively.

Figure 2 shows the variation of the Lindemann index during the heating–cooling cycle of aluminum. The results were obtained over an average of 10 individual simulations, with a heating/cooling rate corresponding to 2 K/ps. The rapid increase of the Lindemann index at *T* > 1050 K during the heating process indicates a solid–liquid transition. The drop in the Lindemann index during the cooling process corresponds to the transition from the metastable liquid to the crystalline solid, and the transition is less abrupt than the solid–liquid, and takes place over a temperature range of ∼50 K. The cooling rate has a substantial impact on determining the location of the supercooled liquid–solid transition temperature,^55^ thereby highlighting the considerable uncertainty of the empirical formula 6 in estimating melting temperatures. From our calculations, we infer that the solid–liquid and supercooled liquid–solid transitions take place at *T*_1_ = 1040 ± 10 K and *T*_2_ = 590 ± 50 K, respectively. Using these temperatures and eq 6, we estimate a melting temperature of 846 ± 50 K.

**Figure 3:**
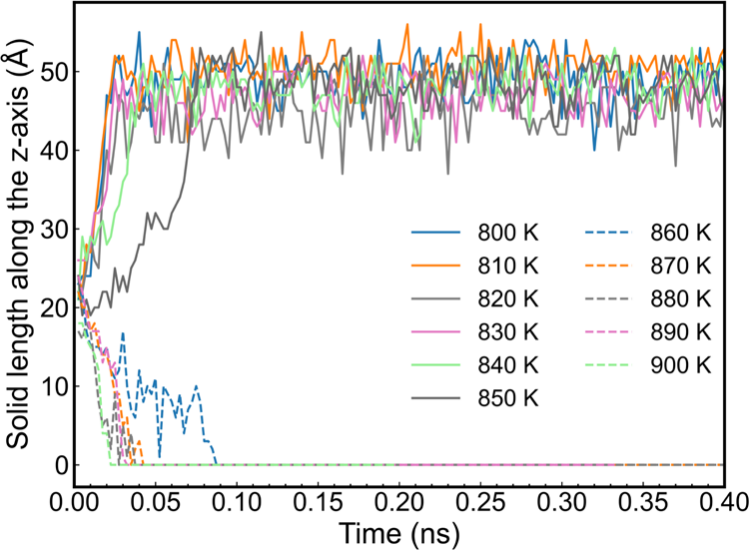
Length of the solid slab along the *z*-axis of the solid–liquid aluminum system as a function of time and temperature. All of the simulations were performed at 1 bar pressure. The solid and dashed lines denote the freezing and melting processes, respectively.

The calculation provided an estimate of the ReaxFF aluminum melting temperature. To calculate the melting temperature, we built configurations consisting of solid–liquid phases in contact by simulating two explicit liquid–solid interfaces (see Figure 1 for an example of an initial configuration). The stability of the liquid–solid interface was investigated using the direct coexistence method in the temperature range of 800–900 K. The simulations were performed in the NPT ensemble at 1 bar pressure using anisotropic barostats. One barostat acted in the direction perpendicular to the solid–liquid interface plane (*z*), and it was decoupled from the parallel directions (*x*, *y*). The length of the crystal, *l*, was calculated to monitor the stability of the liquid–solid interface (see Figure 3) and to follow the crystallization/melting processes. A rapid increase in *l* indicates the formation of a solid phase and takes place at temperatures below the thermodynamic melting temperature. Similarly, a decrease in *l* indicates that the simulation temperature is above the melting temperature (see Figure 3). The value of *l* remains stable at the melting temperature. Hence, the direct coexistence method provides a route to accurately calculate the melting temperature by scanning the temperature around the previously guessed melting temperature of the system. The aluminum system quickly reaches equilibrium, converging to stable solids or liquids at all temperatures within 0.1 ns (Figure 3). This observation agrees with previous studies by Klimanova et al., who investigated the melting of aluminum using the EAM force field.^56^ The rapid convergence emerges from the fast crystal growth rate of metals, an observation connected to a barrierless crystal growth process.^18^ This fast growth also enabled the observation of complete crystallization in our simulations. Moreover, the convergence time increased as the system approached the melting temperature during the melting or solidification. The interface moving rates can be directly calculated from the time trajectories, with velocities ranging from 100 to 200 m/s observed across the temperature range of 850–800 K, respectively. This ultrafast velocity exhibits a magnitude similar to that observed in the aluminum system simulated using the embedded-atom (EAM) method potential model.^18^

**Figure 4:**
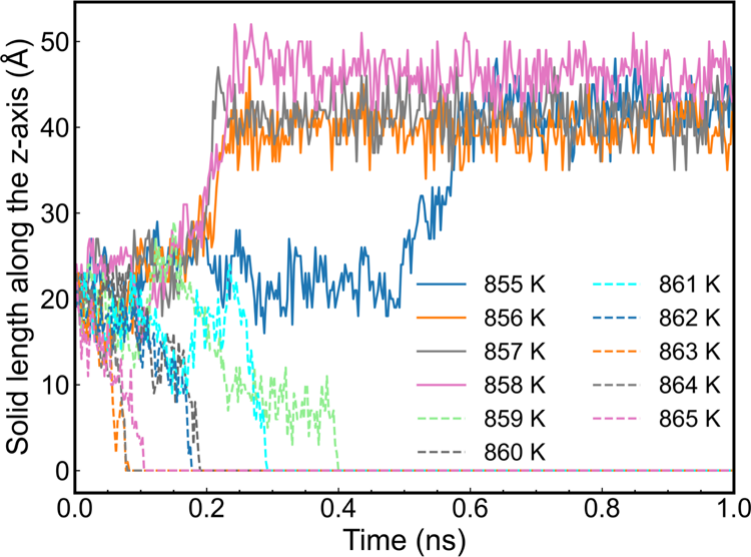
Time dependence of the length of the solid slab along the *z*-axis for aluminum direct coexistence simulations using a 1 K scan step. The solid line denotes solidification, and the dashed line denotes melting.

Due to the barrierless nature of metal crystallization, slight temperature differences of ∼1 K are enough to drive the system to a phase change within a 1 ns simulation time scale. This fact significantly reduces the uncertainty in the computation of the melting temperature using the direct coexistence method. Hence, further simulations at 1 K temperature intervals were conducted (Figure 4). We targeted the interval from 855 to 865 K. The initial configurations for a 1 K scan step were built using the 860 K configuration, and only the thermostat was adjusted. The melting point was defined as the average temperature corresponding to the most stable conditions for melting and crystallization (Figure 4). Using this approach, we estimate a melting point for ReaxFF aluminum of 858 ± 2 K. We found that the temperature difference between conditions (855 and 859 K) is 4 K, rather than the desired 1 K. This observation might be attributed to the relative small length scale of the simulation system with a box length of 3 nm. In our current NVT ensemble, we observed temperature fluctuations of up to 3 K, potentially leading to phase transitions. The additional uncertainty could be mitigated by concurrently considering melting and crystallization. Finally, the melting point was determined to be 858 ± 2 K based on the averaged results of 10 replica simulations, with temperatures corresponding to the slowest rates of crystallization and melting computed at 857.6 ± 1.9 and 859.0 ± 0.4 K, respectively. This temperature is significantly different from the one estimated using eq 6, 846 ± 50 K, which also has a large uncertainty. Otherwise, our melting temperature from direct coexistence, 858 ± 2 K, is in good agreement with the result obtained by Yan et al.,^11^ who reported a value of 860 ± 5 K. The higher uncertainty in their study was attributed to the lack of replica studies, as their primary focus was on the behavior of the liquid aluminum/alumina interface. Additionally, the predicted density is also underestimated with a value of 2.59 kg/m^3^, which is lower than the experimental value of 2.70 kg/m^3^. The predicted Al–Al nearest-neighbor distance, 2.84 Å, is slightly larger than the crystallographic value of 2.82 Å.

### Melting Point of Alumina at Standard Pressure

**Figure 5:**
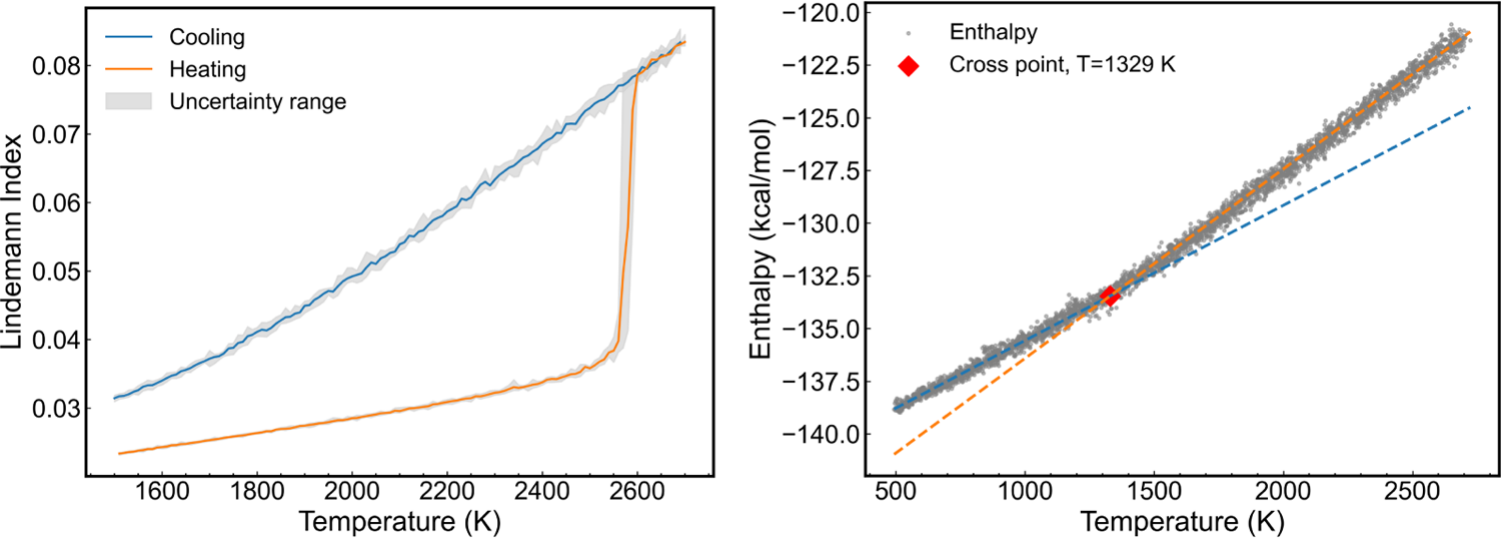
Left: temperature dependence of the Lindemann index of ReaxFF bulk alumina for heating (orange) and cooling (blue) cycles; right: temperature dependence of the enthalpy of bulk Al_2_O_3_ from a cooling process from 2700 to 500 K. The linear fittings (orange and blue lines) were used to locate the transition point (red diamond). The simulations were performed at 1 bar pressure.

We extended the analysis described above to alumina. The determination of the melting point of alumina proved to be more complex. We performed simulations of bulk alumina by superheating/supercooling solid/liquid alumina systems, monitoring the evolution of the system using the Lindemann index (see Figure 5, left). The alumina heating process is similar to that reported for aluminum (Figure 2), and we observe a clear transition in the Lindemann index at a well-defined temperature of 2550 K. In contrast, for supercooled conditions, the Lindemann index does not provide an indication of a transition to the solid phase. Instead, we observe a slope change of the system enthalpy at 1329 K (Figure 5, right), which we assign to a glass-transition temperature.

**Figure 6:**
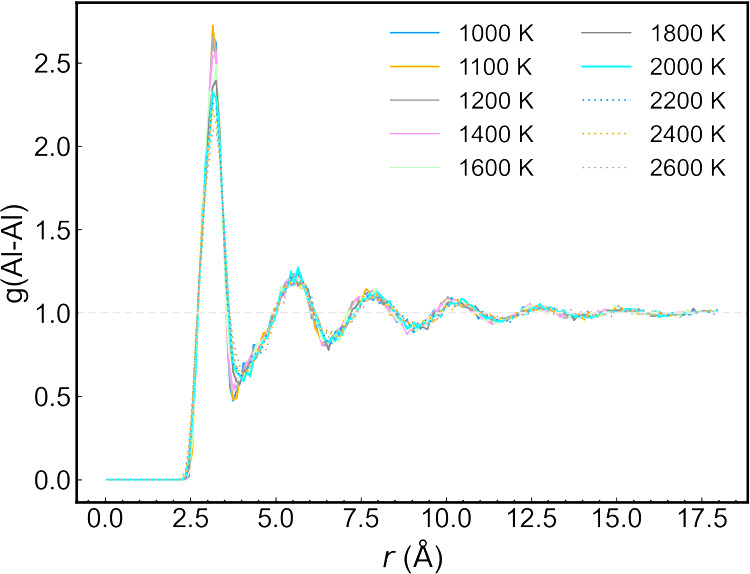
Al–Al RDFs for alumina at different temperatures during the cooling process.

To further investigate the metastable liquid generated during the supercooling process, we computed the radial distribution function (RDF) at different temperatures (Figure 6). The RDFs vary little with temperature, even below 1329 K, where we observe a slope change in the enthalpy (Figure 5, right). This result indicates that the change in slope is not connected to the formation of a crystalline structure. We attribute this transformation to the excessively fast cooling rate of 2 K/ps, which was limited by the simulation speed. Due to the absence of a supercooled liquid–solid transition, the use of eq 6, as was done for aluminum, is not straightforward. Using the Lindemann index jumps on the heating curve and glass-transition point, we find *T*_1_ = 2550 ± 10 K and *T*_2_ = 1329 ± 10 K from the enthalpy slope change, giving an initial guess for the melting temperature of alumina of 2038 ± 10 K, according to eq 6.

**Figure 7:**
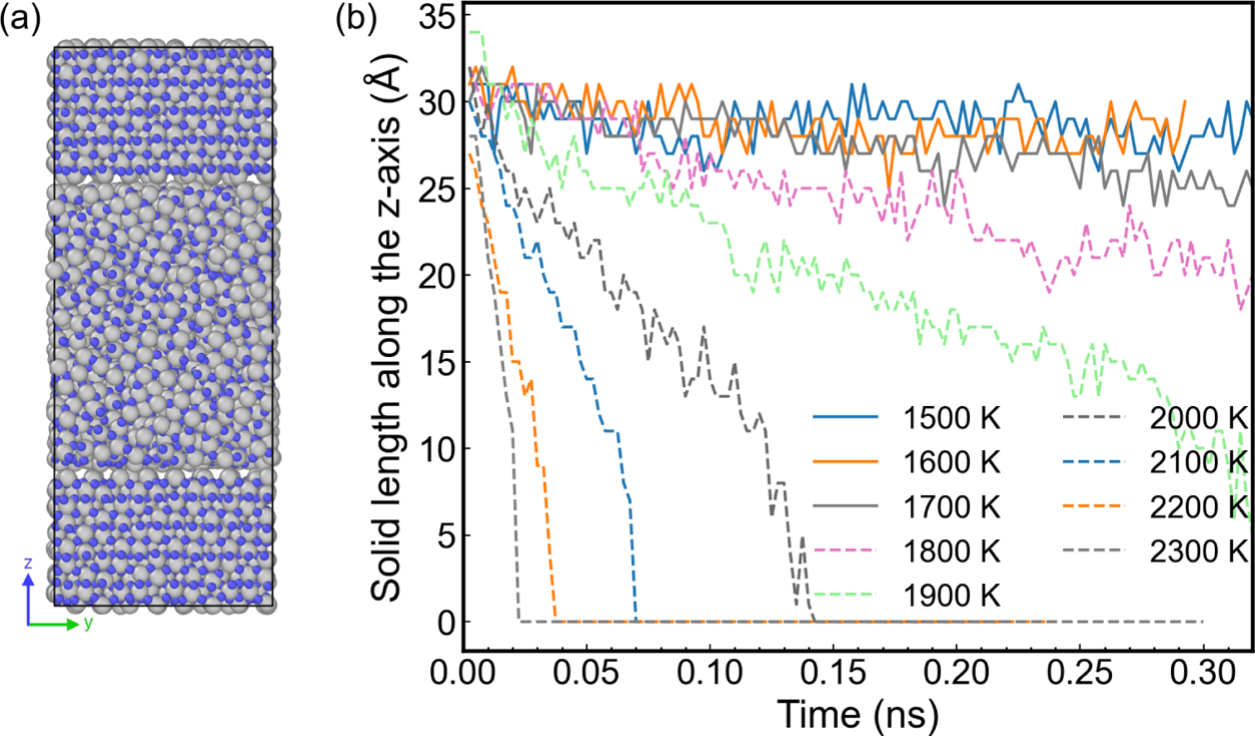
Snapshot of the initial coexistence configuration; gray and blue spheres represent aluminum and oxygen atoms, respectively (a). Variation of the alumina solid phase length along the *z*-axis using the direct coexistence method (b). The lines represent results for different temperatures. Dashed lines represent states that transition to the liquid phase, and full lines state where the solid and liquid phases do not evolve over time.

We performed direct coexistence simulations of alumina, targeting temperatures around the value of 2038 K estimated above (see Figure 7b). A snapshot of a typical configuration is shown in Figure 7a. Our simulation clearly shows evidence for melting at high temperatures *T* > 2000 K for the simulation time scale investigated here, 0.2 ns. However, at lower temperatures, we find a “stagnation” of the length of the solid slab and no evidence of the system evolving to a crystal phase. The lack of transition to the solid phase can be attributed to an energy barrier for crystal growth, which at low temperatures leads to a negligible crystal growth rate, for the time scales investigated here, which are limited by the ReaxFF setup. The results above indicate that the direct coexistence method does not provide a practical approach to obtaining the melting temperature of ReaxFF alumina. To circumvent this problem, we investigated the coexistence of alumina at high-pressure conditions.

### Melting Points of Alumina at High Pressures

The results above show that the direct coexistence method cannot be used to calculate the melting temperature of alumina under standard pressure conditions. If energy barriers are responsible for the stagnation of the crystal growth process for our simulation time scales, these barriers could be overcome at higher melting temperatures.

Following the Clapeyron equation, the melting temperature of alumina will increase with the pressure for a positive change of volume upon melting. Hence, at sufficiently high pressure, one could potentially speed up the crystal growth dynamics, so that it is captured in simulations spanning nanoseconds time scales. Furthermore, the temperature difference between the supercooled liquid–solid transition and the glass transition at higher melting temperatures could be sufficiently different to observe the liquid–solid transition. In support of this idea, we note that Adrjanowicz et al.^33^ investigated the effect of high pressure on the crystallization kinetics of dimethyl phthalate. Their experiments revealed that a high pressure accelerated the crystallization process. They reported an increase in the temperature difference between the melting and glass-transition temperatures of 36 K per GPa. The temperature elevation due to pressure would amplify both the diffusive term and the chemical potential difference in eq 7, thereby increasing the crystal growth rate.

**Figure 8:**
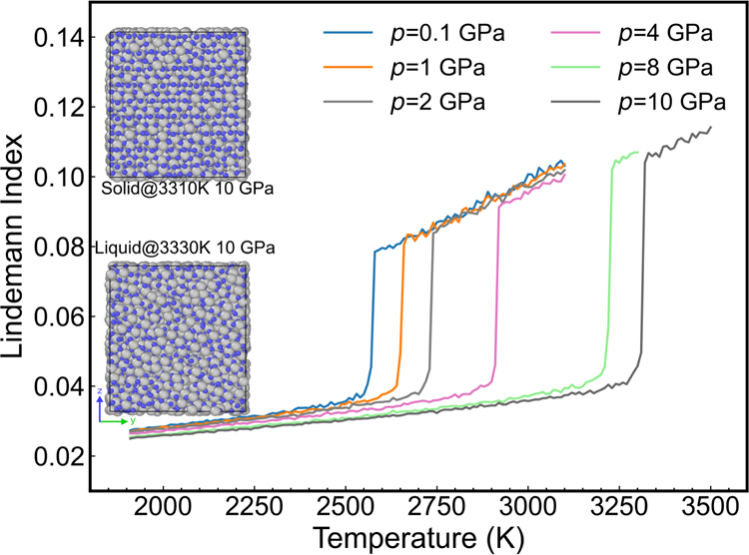
Dependence of the Lindemann index of bulk alumina with the temperature as a function of the temperature. The “jump” in the Lindemann index appears when the superheated solid transitions to liquid alumina at the corresponding pressure. The heating rate is in all cases 2 K/fs. The snapshots show the microstructural changes as the Lindemann index “jumps”, where gray and blue spheres represent aluminum and oxygen atoms, respectively.

We performed a series of alumina coexistence simulations targeting a wide pressure range between 0.1 and 10 GPa to test this hypothesis. The maximum pressure of 10 GPa in the current simulation was found sufficiently high to observe crystal growth in subnanosecond time scales while kept far from the pressure-induced change threshold of the crystal structure, corresponding to an experimental pressure *p* > 116 GPa for α-Al_2_O_3_.^57^ Pressures in the GPa range are common in geophysics, and the kinetics of crystal growth play a critical role. The Lindemann indices were calculated to monitor the onset of the phase change and to estimate the pressure dependence of the phase transition temperature (see Figure 8). The temperature of the superheated solid–liquid transition increases with the pressure. This can be seen in a shift of the Lindemann index “jump” to higher temperatures as the pressure increases.

**Figure 9:**
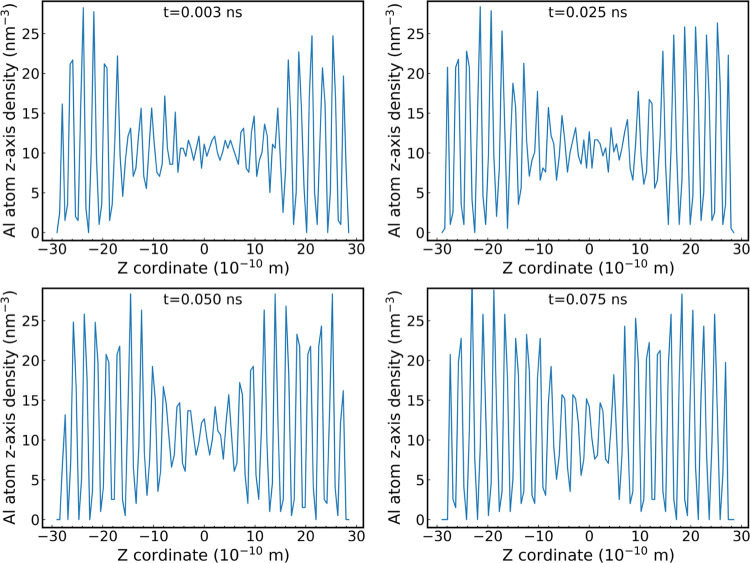
Aluminum atom density profile in the alumina direct coexistence system at 10 GPa and 2400 K. The four panels show different stages of the crystal growth process.

To calculate the thermodynamic melting temperature of ReaxFF alumina, we performed simulations using the direct coexistence method by putting together alumina crystal and liquid phases. The fastest crystal growth rate was observed at 2300 K and 10 GPa. Figure 9 shows the average density profile of aluminum atoms in alumina along the *z*-axis of the simulation box and at different simulation times. Our results clearly show evidence of crystal growth toward the center of the simulation cell, indicating that the crystallization process is taking place.

We quantified the impact of the temperature and pressure on the interface kinetics by calculating the interface front speed *u* using the equation
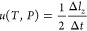
8where *u* is the interface speed, *l*_*z*_ is the width of the solid slab along the *z*-axis for the liquid part, and the 1/2 factor accounts for the presence of two interfaces.

**Figure 10:**
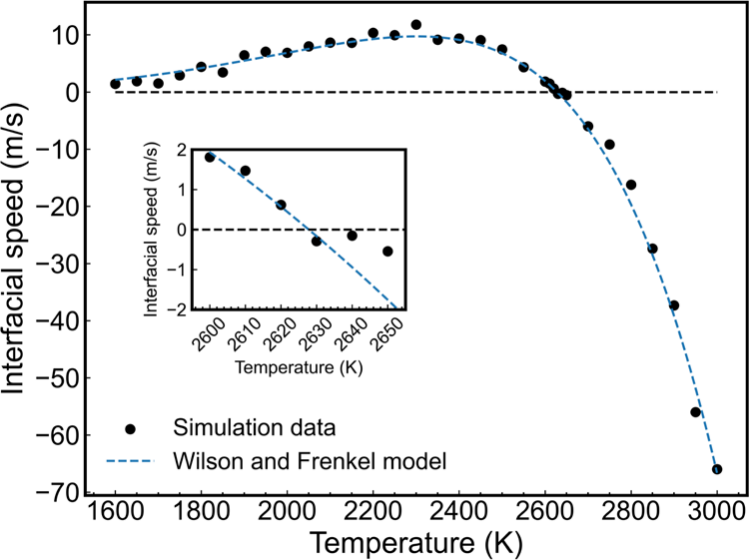
Interfacial speed for the alumina system under direct coexistence conditions and 10 GPa. The dashed line represents the fitting from the Wilson and Frenkel model, eq 7.^51,52^ Negative speeds correspond to melting, and positive speeds correspond to freezing.

We show in Figure 10 the interfacial speed of alumina in the direct coexistence system at 10 GPa for various temperatures. The Wilson and Frenkel eq 7 provides a good fit of our interfacial speeds with temperature. The agreement between our data and eq 7 suggests that the activation barriers for diffusion and the thermodynamic force for the liquid–solid transition defined the speed *u* in different temperature regimes. The melting temperature, derived from the fitting parameters of formula 7, was found to be 2627 ± 10 K. In the vicinity of the melting temperature region, the interfacial velocity features a symmetry against the *x* axis, which could be attributed to the dominant driving force being the thermal difference between the system temperature and the melting temperature. The interface moving rate decreased as the temperature decreased, a result that we attribute to a slowing down of the diffusion of the liquid, possibly connected to the onset of vitrification.

**Figure 11:**
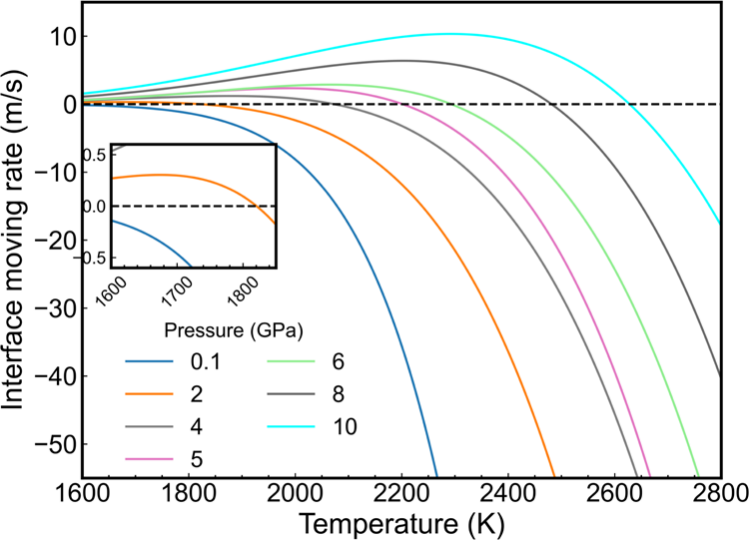
Interfacial speed of alumina obtained from direct coexistence simulations at different pressures. The dashed horizontal line with a zero value is used to identify the melting points.

We performed additional simulations of the interfacial speed at different pressures in the range of 0.1–10 GPa and covering a wide temperature range between 1600 and 2800 K. The results are represented in Figure 11. The pressure clearly influences the phase transition temperature (Figure 8). First, the temperature at which the system features zero interfacial speed, i.e., the melting temperature, decreases with pressure, as expected. Second, at pressures lower than 2 GPa, we failed to observe the freezing process and only found negative values for the interfacial speed *u*. Finally, the maximum interfacial speed decreases from 10.32 m/s at 2292 K, 10 GPa to 0.30 m/s at 1674 K, 2 GPa. These speeds are 2–3 orders of magnitude lower than those we obtain for aluminum (see Figure 4), which reached speeds of ∼100 m/s at standard pressure.

The small but not negligible interfacial speeds obtained in our metal oxide crystallization process at high pressure provide a route to quantifying the melting point of alumina. The equilibrium Al–Al interatomic distance for α-Al_2_O_3_ is 2.74 Å at standard conditions.^37^ Ideally, to observe the crystallization of a single layer within a 1 ns simulation, *u* should exceed 0.27 m/s. The relaxing time for freezing under high-temperature conditions is negligible, typically within the femtosecond range.^58^ Simulations under 2 GPa with an estimated maximum speed rate of 0.14 m/s represent the minimum practical observable condition for freezing within 1 ns.

**Figure 12:**
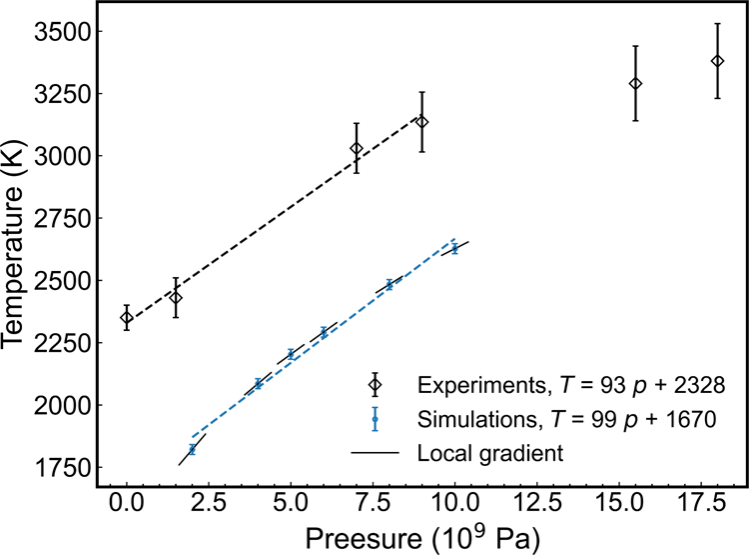
Pressure dependence of the melting temperatures of alumina obtained from direct coexistence simulations. The dashed lines represent linear regressions using pressure values <10 GPa, and the solid lines represent the local gradients calculated using Clapeyron eq 9. Experiment data is taken from ref (59)

Although extending the simulation time can decrease the minimum observable growth rate, for instance, the melting simulation lasting up to microseconds for silica,^30^ increasing the simulation pressure to enhance the growth rate, and extrapolating toward lower-pressure levels may provide a more effective computational strategy, especially considering the good linear pressure dependency of the melting temperature under moderate pressures (see Figure 12). We used this fact to extrapolate the atmospheric melting temperature of alumina, which was estimated to be 1670 ± 10 K at 1 bar pressure, where the uncertainty from the 10 K scan step was for the coexistence system. Based on the Clapeyron equation, the local gradient of the pressure dependency of melting temperature can be obtained using the Clapeyron equation
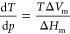
9where Δ*V*_m_ and Δ*H*_m_ are the molar volume and enthalpy of melting at temperature *T* and pressure *p*, respectively. In this article, the values of Δ*V*_m_ and Δ*H*_m_ were directly calculated from bulk phase simulations of the solid and liquid phases at the corresponding temperature and pressure.

The melting temperature–pressure curve (see Figure 12) features a clear linear dependence with pressure for pressures <10 GPa. Our simulation shows a high level of consistency in both the average gradient d*T*/d*p*. Specifically, the simulation yields d*T*/d*p* = 99 K/GPa, slightly higher than the experimental value of 93 K/GPa.^59^ Additionally, the local gradient of the simulation decreases with increasing pressure, ranging from d*T*/d*p* = 154 K/GPa at 2 GPa to 67 K/GPa at 10 GPa. Based on a linear extrapolation, our estimate of the standard melting temperature of ReaxFF alumina is 1670 K, notably lower, 28%, than the experimental result.^13^ This result contrasts with the much better prediction of the ReaxFF for aluminum, where the standard melting temperature is underestimated by 8%. These results indicate that there is room to improve the accuracy of the metal oxide ReaxFF. Similarly to aluminum, the predicted density of alumina is also underestimated, with a value of 3.84 kg/m^3^, which is lower than the experimental value of 3.99 kg/m^3^. The predicted Al–O distance of 1.94 Å is slightly larger than the crystallographic value of 1.92 Å.

The activation energy *Q* can be determined from eq 7 by considering the maximum speed, namely, , where the *T*_max_ corresponds to the temperature with the maximum of velocity. The activation energy was estimated using the following equation
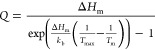
10

Notably, the fitting parameter *f*_0_ does not appear in eq 10 since the maximum emerges from a competition between the diffusion and chemical potential exponential terms.

We are unaware of previous theoretical analyses addressing the dependence of *Q* on pressure in the case of alumina. Hence, our work provides simulation evidence that the activation energy *Q* changes linearly in the high-pressure regime for a prototypical metal oxide material. Equation 10 may provide a reference to rationalize the linear dependence. In that equation, *Q* is expressed as a function of the enthalpy, (Δ*H*_m_), and (*T*_max_ – *T*_m_), i.e., the temperature difference between the maximum interface moving speed and the melting point. In our calculations, we observed a linear pressure dependence of *T*_m_. This result agrees with the experimental data reported in ref (59) (see Figure 12), where the melting temperature features a linear dependence on the pressure, which we use to support our extrapolation to lower pressures.

Interestingly, previous studies of tetrahedral liquids, water, reported a non-Arrhenius behavior of the diffusion with temperature.^60^ This behavior indicates a temperature-dependent activation barrier for diffusion. Furthermore, these authors reported simulations supporting an activation energy that decreases with increasing pressure. This result is consistent with experiments in confined water.^61^ Also, the analyses reported in ref (60) showed a linear dependence with pressure, which they rationalized using a mean-field analysis. Establishing the microscopic mechanism determining the linear dependence of *Q* with *P* observed in our work will require additional investigation, which should be an important topic for future works.

**Figure 13:**
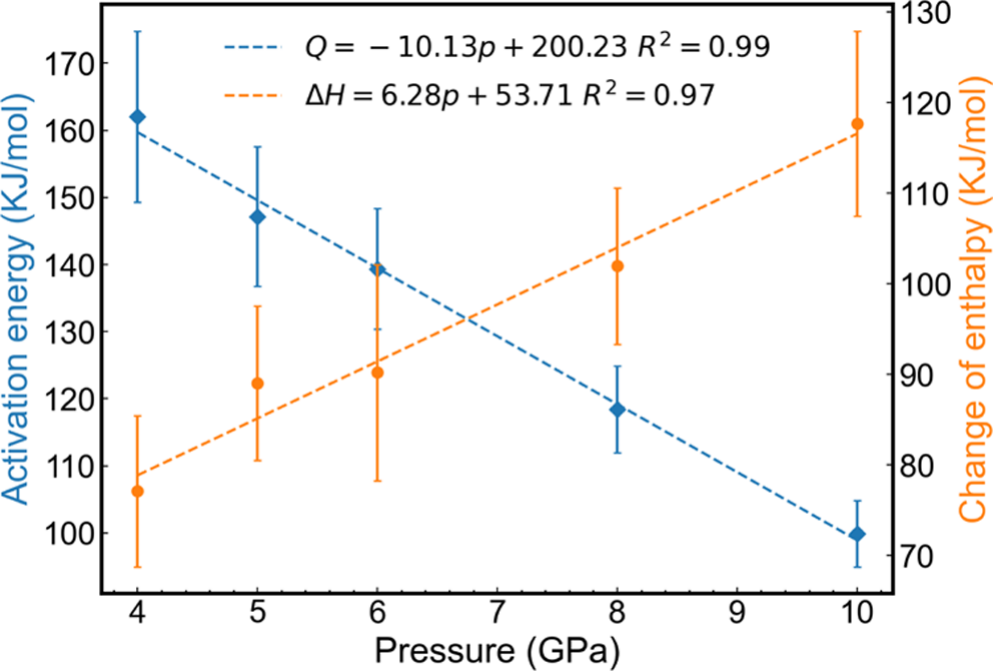
Pressure dependence of the activation energy and the enthalpy of melting. The uncertainty of *Q* is assumed based on the 10 K uncertainty of *T*_max_. The result at 2 GPa is excluded from the analysis due to the inability to accurately determine its corresponding *T*_max_.

Based on the discussion above and the linear dependence at high pressure, the activation energy *Q* at atmosphere pressure can be determined to be 200.23 kJ/mol through a linear exploitation from the results of higher pressures (see Figure 13).

Similarly, the standard enthalpy of melting is 53.71 kJ/mol with the same method. We attribute the discrepancy between this value and the experimental value of 111.1 kJ/mol^62^ to the lower melting temperature in this simulation (1670 K compared to 2326 K). At a pressure of 6.6 GPa, the *T*_m_ for simulation is 2326 K, and the Δ*H*_m_ would be 95.16 kJ/mol, with a 14% difference from the experimental value.

## Conclusions and Final Remarks

We have investigated the crystal growth and melting points of metals (aluminum) and metal oxides (alumina) using the ReaxFF force field and molecular dynamics simulations and introduced an approach to quantify the standard melting temperature of oxides using direct coexistence simulations. Direct coexistence simulations with explicit liquid–solid interfaces provide a viable route to quantify the crystal growth dynamics and melting points of metals modeled with ReaxFF. Our results support the view that crystal growth in metals is a barrierless process and, therefore, very fast at molecular scales. This makes ReaxFF and molecular dynamics simulation a valid approach to investigate these materials. For aluminum, the Lindemann index was used to follow the solid–liquid transition. Based on direct coexistence simulations, we predict a melting point of 858 ± 2 K, in reasonable agreement with experimental data, 933 K. ReaxFF is valued for its transferability across similar systems, such as aluminum, alumina, and their interfaces, using a consistent parameter set.^7,14^ However, its accuracy relies on the quality of its training data, which was based primarily on metal clusters. This could lead to discrepancies in the prediction of melting temperatures. Additionally, certain terms related to long-range interactions or many-body effects may not be well-parameterized, contributing to discrepancies in predicted melting temperatures.

The investigation of crystal growth and quantification of the standard melting temperature of ReaxFF metal oxides (alumina) using direct coexistence methods presents unique challenges due to the tendency of the oxide to form glass rather than crystalline structures in the typical time scales used in the cooling process simulated with molecular dynamics. We circumvented this problem by targeting high-pressure conditions, where the melting temperature increases relative to the standard pressure. Our simulations show that the crystal growth dynamics speed up considerably at pressures >2 GPa, making it possible to model the kinetics of crystal growth of alumina using nanosecond time scales typical of reactive force field simulations. We also found that the melting temperature features a strong linear dependence on the pressure, consistent with experimental observations. This fact can be used to quantify the standard metal temperature of the metal oxide.

Our approach provides a route to quantify the crystal growth dynamics and melting of metals and oxides modeled with reactive force fields. The results presented in this paper serve as a reference for refining current computational models of these materials and for modeling the thermodynamic behavior of metal oxides under nonstandard conditions.

## Supporting Information Available

Replicas of aluminum coexistence simulation with a 1 K scan step and pressure dependence of the superheated transition temperature (PDF)
Lammps input files (ZIP)
