# Supplementary material for: Melting Point and Crystal Growth Kinetics of Metals and Metal Oxides Using Reactive Force Fields: The Case of Aluminum and Alumina
Source: J Chem Theory Comput. 2024 Sep 5;20(18):8190–201. doi: 10.1021/acs.jctc.4c00628 (PMC11428160; doi:10.1021/acs.jctc.4c00628)
Supplement: Supplementary file 1 — ct4c00628_si_001.pdf [file ct4c00628_si_001.pdf]

Supporting Information: Melting Point and Crystal Growth Kinetics of Metals and Metal Oxides using Reactive Forcefields: the case of Aluminum and Alumina

Hao Zhao[†,‡] and Fernando Bresme[*,†]

†*Department of Chemistry, Molecular Sciences Research Hub, Imperial College, London, W12 0BZ, United Kingdom*

‡*State Key Laboratory of Multiphase Flow in Power Engineering, Xi'an Jiaotong University, Xi'an, Shaanxi, 710049, China*

E-mail: f.bresme@imperial.ac.uk

CONTENTS

1. Replicas of Aluminum coexistence simulation with 1 K scan step.

2. Pressure-dependence of the superheated transition temperature.

SUPPLEMENTAL INFORMATION

Figure S2 shows the pressure dependence of the temperature at which we observe the "jump" in the Lindemann index in the heating cycle. Our temperature-pressure plot features a slope similar to the one observed in the experimental melting temperature/pressure line reported in

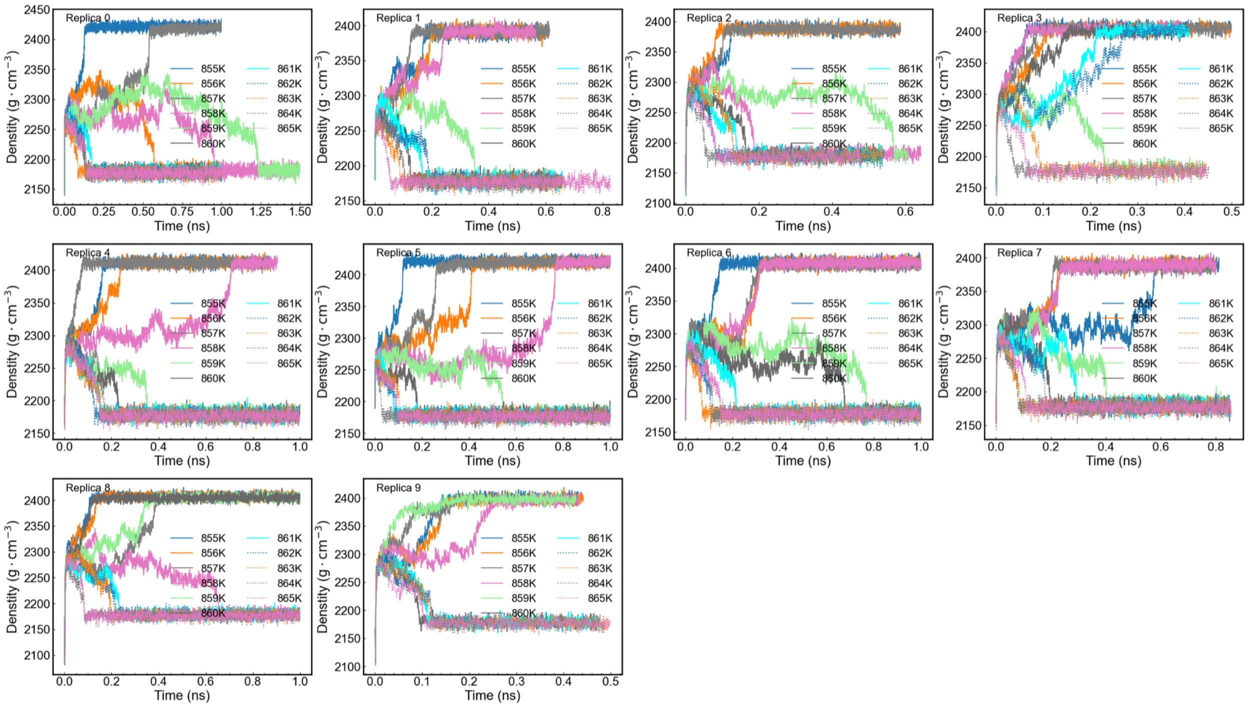

Figure S1: Replicas of Aluminum coexistence simulation with 1 K scan step.

reference.[S1] We note that the temperature at which we observe the “jump” is not the melting temperature since the data correspond to the transition of a superheated solid and not to the thermodynamic solid-liquid phase transition. Hence, these data cannot be compared directly with the experimental melting temperatures,

References

(S1) Shen, G.; Lazor, P. Measurement of melting temperatures of some minerals under lower mantle pressures. *Journal of Geophysical Research: Solid Earth* **1995**, *100*, 17699–17713.

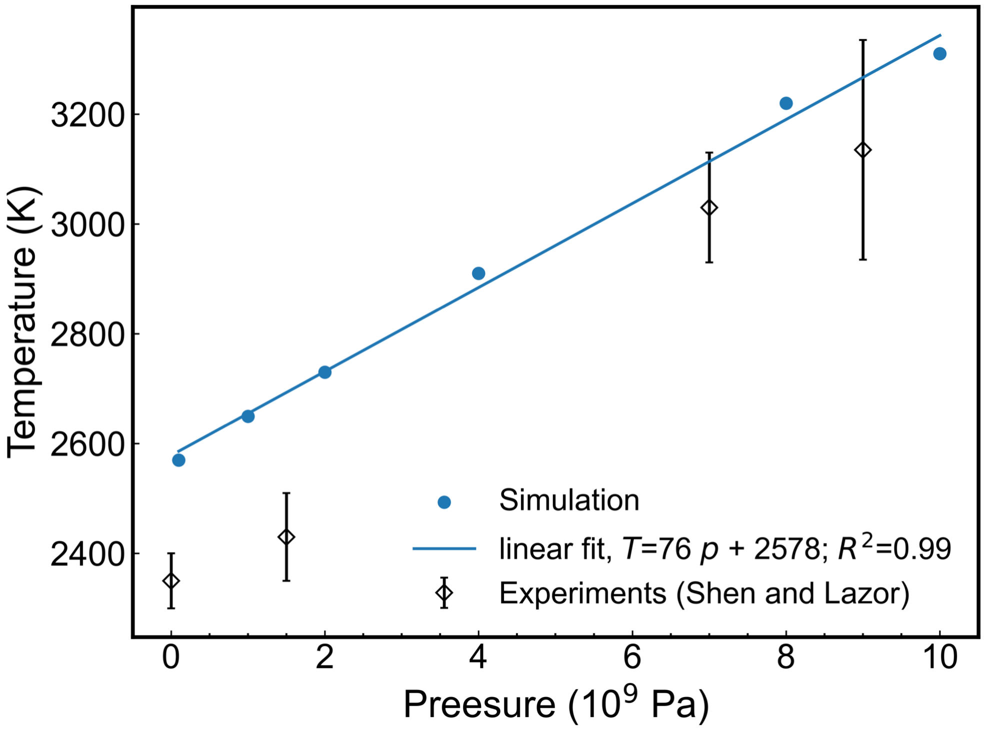

Figure S2: Pressure-dependence of the temperature corresponding to the "jump" in the Lindeman index(blue symbols) . Experimental melting temperature of alumina as a function of pressure (black symbols) . Data taken from reference.[S1]
